# Coding algorithms for defining Charlson and Elixhauser co-morbidities in Read-coded databases

**DOI:** 10.1186/s12874-019-0753-5

**Published:** 2019-06-06

**Authors:** David Metcalfe, James Masters, Antonella Delmestri, Andrew Judge, Daniel Perry, Cheryl Zogg, Belinda Gabbe, Matthew Costa

**Affiliations:** 1 Oxford Trauma, Kadoorie Centre for Critical Care Research and Education, Nuffield Department of Orthopaedics, Rheumatology and Musculoskeletal Sciences (NDORMS), John Radcliffe Hospital, Headley Way, Oxford, OX3 9BU UK; 2 Centre for Statistics in Medicine, NDORMS, Nuffield Orthopaedic Centre, University of Oxford, Windmill Road, Oxford, OX3 7LD UK; 3 Musculoskeletal Research Unit, Translational Health Sciences, Bristol Medical School, University of Bristol, Learning and Research Building, Level 1, Southmead Hospital, Bristol, BS10 5NB UK; 4 National Institute for Health Research Bristol Biomedical Research Centre (NIHR Bristol BRC), University Hospitals Bristol NHS Foundation Trust, University of Bristol, Southmead Hospital, Bristol, BS10 5NB UK; 5 MRC Lifecourse Epidemiology Unit, University of Southampton, Southampton General Hospital, Southampton, SO16 6YD UK; 6 Yale School of Medicine, 333 Cedar Street, New Haven, CT 06510 USA; 7 School of Public Health and Preventive Medicine, Monash University, Level 3, 553 St Kilda Road, Melbourne, VIC 3004 Australia

**Keywords:** Comorbidity, Read codes, Charlson, Elixhauser

## Abstract

**Background:**

Comorbidity measures, such as the Charlson Comorbidity Index (CCI) and Elixhauser Method (EM), are frequently used for risk-adjustment by healthcare researchers. This study sought to create CCI and EM lists of Read codes, which are standard terminology used in some large primary care databases. It also aimed to describe and compare the predictive properties of the CCI and EM amongst patients with hip fracture (and matched controls) in a large primary care administrative dataset.

**Methods:**

Two researchers independently screened 111,929 individual Read codes to populate the 17 CCI and 31 EM comorbidity categories. Patients with hip fractures were identified (together with age- and sex-matched controls) from UK primary care practices participating in the Clinical Practice Research Datalink (CPRD). The predictive properties of both comorbidity measures were explored in hip fracture and control populations using logistic regression models fitted with 30- and 365-day mortality as the dependent variables together with tests of equality for Receiver Operating Characteristic (ROC) curves.

**Results:**

There were 5832 CCI and 7156 EM comorbidity codes. The EM improved the ability of a logistic regression model (using age and sex as covariables) to predict 30-day mortality (AUROC 0.744 versus 0.686). The EM alone also outperformed the CCI (0.696 versus 0.601). Capturing comorbidities over a prolonged period only modestly improved the predictive value of either index: EM 1-year look-back 0.645 versus 5-year 0.676 versus complete record 0.695 and CCI 0.574 versus 0.591 versus 0.605.

**Conclusions:**

The comorbidity code lists may be used by future researchers to calculate CCI and EM using records from Read coded databases. The EM is preferable to the CCI but only marginal gains should be expected from incorporating comorbidities over a period longer than 1 year.

**Electronic supplementary material:**

The online version of this article (10.1186/s12874-019-0753-5) contains supplementary material, which is available to authorized users.

## Background

The comparison of patient outcomes between healthcare providers requires effective risk adjustment for patient characteristics. In particular, comorbidities are important predictors of outcome^1 2^. Comorbidity summary measures have been developed to help classify patients according to their overall disease burden [1–4].

The most commonly used summary measure is the Charlson Comorbidity Index (CCI) [4]. Charlson et al. identified 17 diseases that optimally predict one-year mortality when assigned a weight between 1 (e.g. peripheral vascular disease) and 6 (e.g. metastatic cancer) [1]. Although the CCI is commonly used [4] and has been widely validated [5], it was developed in the 1980s and has been criticized as outdated [6]. A number of meta-analyses have found that an alternative summary measure proposed by Elixhauser et al. [2] has superior predictive properties^3 4^. In particular, the Elixhauser Method (EM) predicts mortality more effectively than CCI amongst patients with fractures of the cervical spine [7] and proximal humerus [8]. However, although older adults with hip fractures have a high comorbid disease burden, it is unclear which summary measure optimally predicts mortality in this population. The EM is similar to the CCI (nine categories overlap the two measures: diabetes [uncomplicated and complicated], congestive heart failure, HIV, metastatic cancer, renal disease, chronic pulmonary disease, rheumatic disease, and peripheral vascular disease) but includes almost twice as many diagnostic categories [9].

A number of algorithms have been developed to determine CCI and EM from administrative databases based on ICD-9 [10–12] and ICD-10 [9] diagnostic codes. Although Khan *et al* [13] have developed an algorithm for calculating CCI in Read-coded databases; there is no equivalent translation for EM. This is important because Read codes are used by General Practitioners throughout the United Kingdom National Health Service (NHS) [14] and are the basis on which a number of national primary care datasets have developed. These include the Clinical Practice Research Datalink (CPRD) GOLD [15] and The Health Improvement Network (THIN) [16] databases.

The aims of this study were to: (1) develop coding algorithms for calculating CCI and EM in Read-coded databases, (2) describe the comorbidity characteristics of a hip fracture cohort with matched controls, and (3) compare the predictive properties of the CCI (both original and modified versions) and the EM.

## Methods

### Defining co-morbidity algorithms

**Fig. 1:**
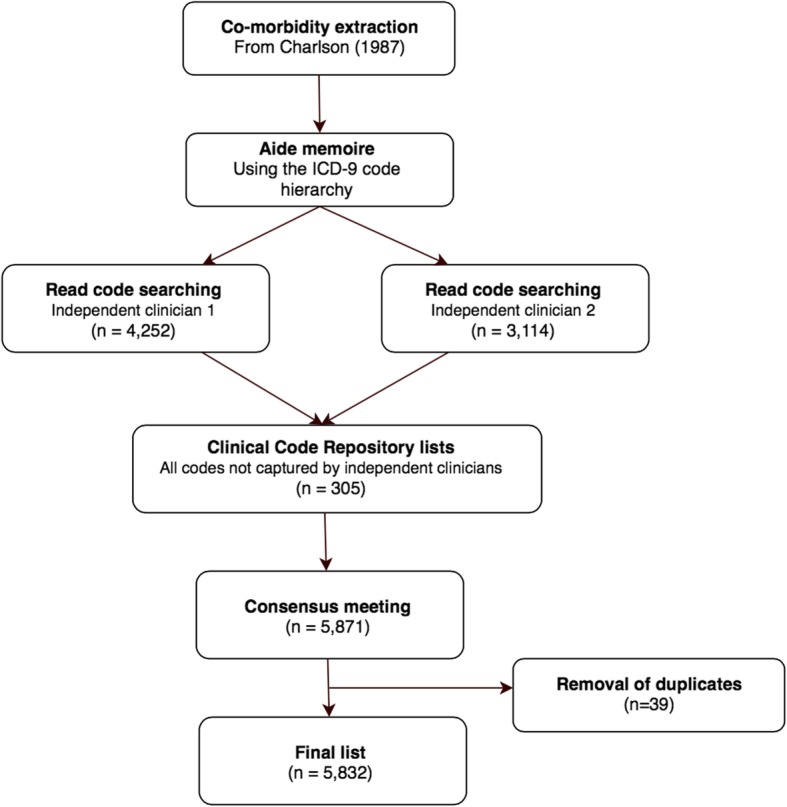
Flow chart showing the exclusion of Charlson co-morbidity Read codes

**Fig. 2:**
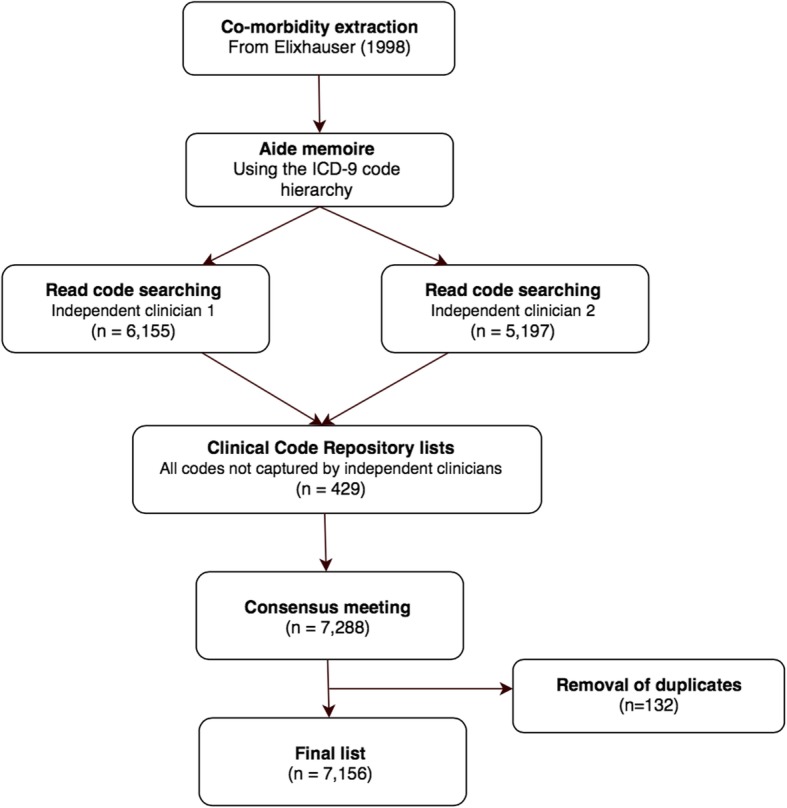
Flow diagram showing the exclusion of Elixhauser co-morbidity Read codes

The multi-step process for selecting comorbidity diagnostic codes is shown by Figs. 1 and 2. First, the 31 co-morbidities defined by Elixhauser et al. [2] and 17 by Charlson et al. [1] were extracted from their original publications. The Charlson paper was supplemented with work by Deyo et al. [11] who previously translated the Charlson co-morbidities into ICD-9-CM codes. Each comorbidity category was presented together with its ICD-9-CM codes and a text interpretation of each code (exploded to show the full hierarchy of sub-codes) from the 6th edition of the International Classification of Diseases, 9th Revision, Clinical Modification (ICD-9-CM) [17]. This step was necessary because many Read terms are unstructured but the ICD-9-CM hierarchy acted as an *aide memoire* for diagnoses that might otherwise be missed. For example, clinicians trying to populate the Charlson category “Any malignancy, including leukaemia and lymphoma” might search for “lymphoma” but could inadvertently omit “mycosis fungoides” (represented by 7 separate Read codes) or “Letterer-Siwe disease” (5 Read codes). However, the researchers would encounter all three codes while working through the “Malignant neoplasm of lymphatic and hematopoietic tissue” chapter of ICD-9-CM. Similarly, even a specialist might search for “myeloid sarcoma” but not think to search for additional Read codes under “chloroma”. They would however find “chloroma” listed under “myeloid sarcoma” in the ICD-9-CM hierarchy.

Two clinicians independently used the exploded ICD-9-CM text codes to search all 111,929 Read terms within the CPRD Medical Dictionary [18]. The general search principles and assumptions agreed by the two code screeners are available in Additional file 3 The result of this process was that each screener developed a list of Read terms that corresponded to the ICD-9-CM codes recommended by Elixhauser [2] and Charlson/Deyo [1, 11]*.*

In addition, the online ClinicalCodes Repository [19] was manually searched for all pre-existing Read code lists that pertained to each comorbidity category. Lists from 12 studies [20–30] were included from the ClinicalCodes Repository in addition to the CCI list previously developed by Khan *et al* [13]*.* The outcome of this process was that between two and six independent Read code lists were generated for each comorbidity category. The two clinicians then resolved discrepancies through discussion and with advice from sub-specialists where appropriate. A single list was generated for each comorbidity measure and duplicate entries deleted. A final logic check was performed by a single clinician.

### Co-morbidity characteristics of a hip fracture cohort

The CPRD is an ongoing primary care database of medical records provided by General Practitioners [15]. It is owned by the Medicines and Healthcare products Regulatory Agency (MHRA) and collects data about more than 11.3 million patients from 674 individual GP practices. Approximately 6.9% of UK residents are currently represented by data in the CPRD and these are broadly representative of the wider UK population. General Practitioners in the UK maintain each patient’s entire healthcare record and should receive correspondence (including Emergency Department correspondence, outpatient clinic letters, and hospital discharge summaries) from secondary care providers. Important events (e.g. hip fracture) and diagnoses (e.g. interstitial lung disease) should therefore be coded into the GP record even if the patient was treated in hospital.

A cohort of patients (cases) were identified from CPRD GOLD practices based on a first ever record of “hip fracture” occurring between 1st January 1999 and 9th October 2013. The diagnostic and procedural codes used to define this hip fracture cohort are presented in Additional file 4 In addition, the patients required at least 3 years up-to-standard (UTS) registration in CPRD GOLD prior to their hip fracture [15]. Age- and sex- matched controls were identified by the CPRD in a 2:1 ratio from patients registered with practices from 1st January 1996 onwards and with at least three preceding years UTS registration.

The specific variables extracted from the CPRD were age, sex, date of hip fracture diagnosis, and individual comorbidities. For the principal analysis, diagnostic codes were extracted from each patient’s entire lifetime primary care record. We also planned sensitivity analyses that confined comorbidities to those recorded within 1- and 5-years of the index hip fracture.

### Validating the predictive properties of the Elixhauser method

The EM was tested against the CCI, using both the 17-item original version by Charlson *et al* [1] and the shorter 12-item modification proposed by Quan *et al* [6]. We planned to report 30- and 365-day mortality. Kaplan-Meier plots were created for death within 365 days by categories of CCI and EM. Logistic regression models were fitted with 30- and 365-day mortality as the dependent variables. The covariables were age (as a continuous variable) and sex, which is consistent with the approach taken by other studies designed to evaluate comorbidity summary measures. The subsequent analyses fitted multivariable logistic regression models with age and sex as well as either CCI or Elixhauser comorbidities as covariables. Stepwise variable selection techniques were not used. The comorbidity summary measures were then layered on top of this base model. Tests of equality for Receiver Operating Characteristic (ROC) areas were undertaken using the *roccomp* [31] module in Stata v.15.0 (College Station, TX, USA). Although summary tables were produced to show the number of EM comorbidities in each group, these were included as separate independent variables within regression models in the manner proposed by Elixhauser et al. [2]. The principal analysis used CCI calculated using the weights originally proposed by Charlson et al. [1]. We reported the predictive properties of the EM and CCI in both diseased (i.e. hip fracture) and non-diseased (age- and sex-matched control) populations. Importantly, we undertook analyses of cases and controls separately and did not plan to utilize a case-control design. In addition, we undertook sensitivity analyses limited to comorbidities documented 1- and 5-years before the index hip fracture as some researchers may find themselves working with cuts of data that are limited in time. The principal analysis used all comorbidies documented at any time in each patient’s complete medical record.

### Information governance

Ethical approval was not sought in line with the latest Governance Arrangements for Research Ethics Committees (GafREC) guidance [32]. Approval to use the data was provided by the Independent Scientific Advisory Committee (ISAC) at the MHRA (ISAC Protocol No. 13_069RA). Personal data was processed under Articles 6 (1)(f) and 9 (1)(f) of the General Data Protection Regulation (EU 2016/6709).

## Results

### Defining an Elixhauser coding algorithm

Figures 1 and 2 show the number of diagnoses identified and eliminated for CCI and EM respectively. The final lists included 5832 individual codes representing CCI comorbidities (Additional file 1) and 7156 EM comorbidities (Additional file 2).

### Comorbidity characteristics of a patient cohort

**Fig. 3:**
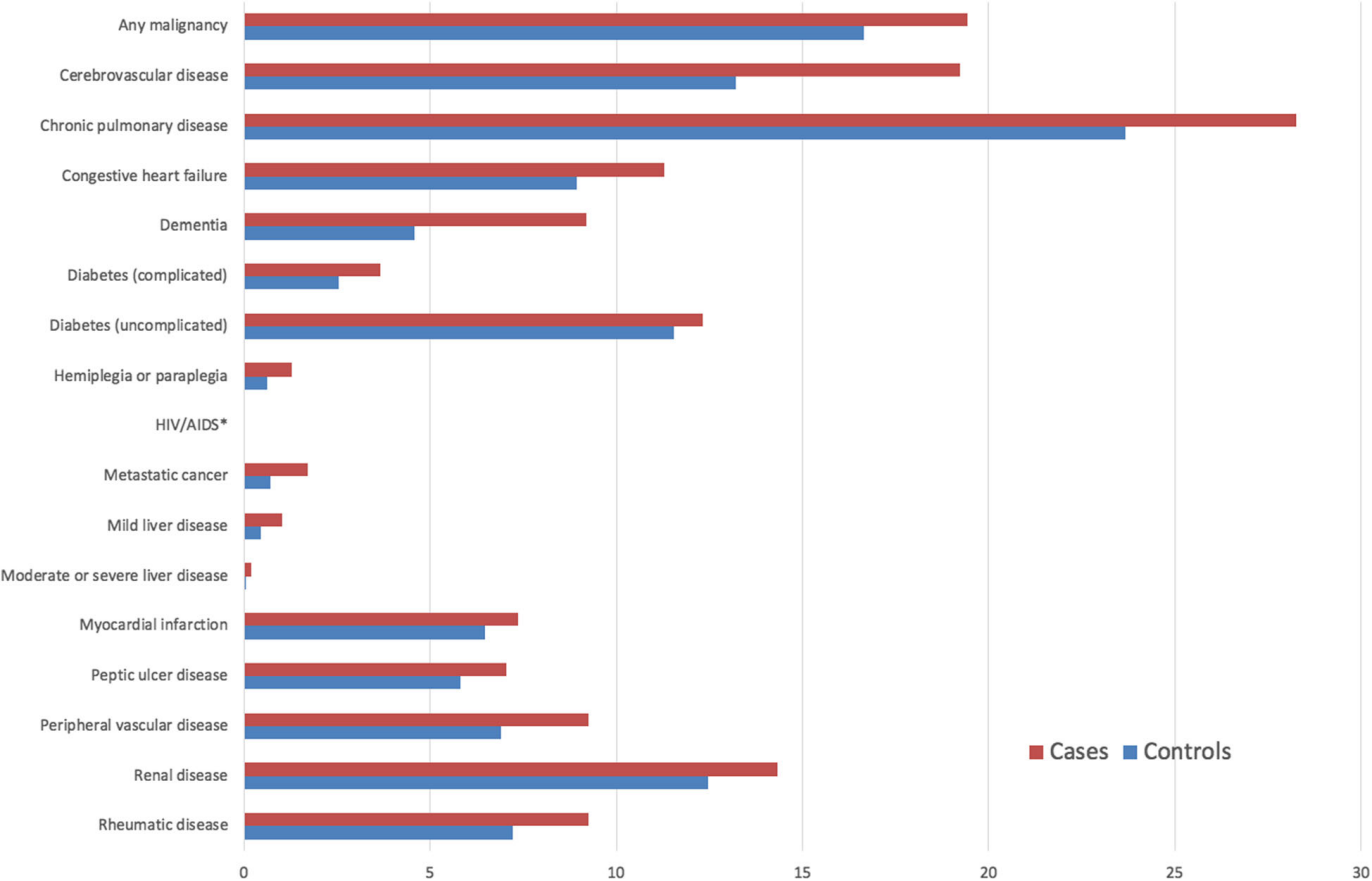
Proportion of cases and controls with each Charlson co-morbidity

**Fig. 4:**
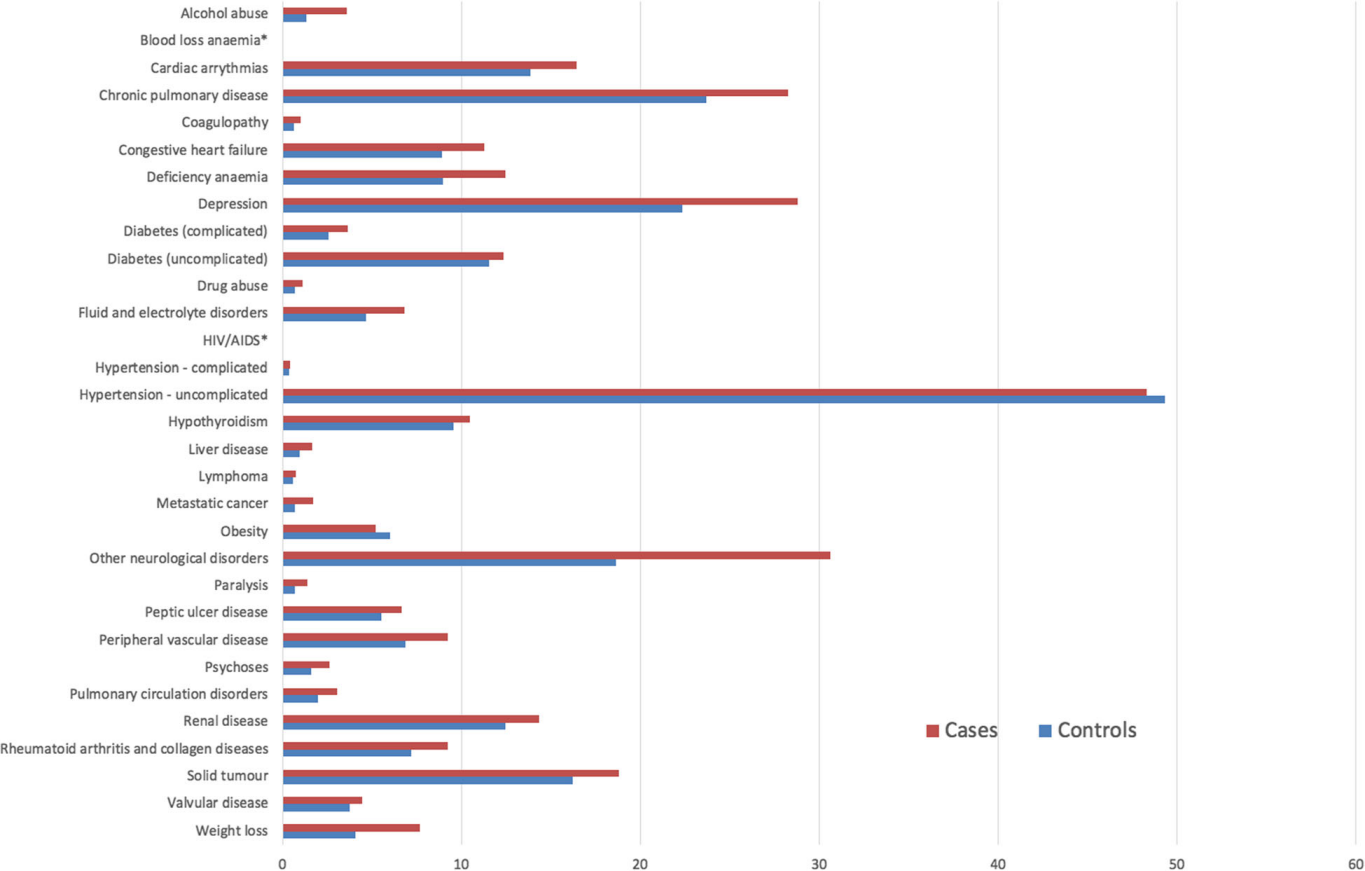
Proportion of cases and controls with each Elixhauser co-morbidity

The linked dataset included 13,974 patients with hip fractures and 26,860 age- and sex-matched controls. The median age across the cohort was 82 years (interquartile range [IQR]: 75–87 years and 75.1% were female. The distribution of comorbidities within the cohort according to Charlson and Elixhauser are shown in Figs. 3 and 4. Table 1 shows that 27.6% of hip fracture patients did not have any CCI co-morbidities recorded; only 9.2% did not have EM conditions recorded. The median CCI was 1 (interquartile range [IQR] 0–3) and EM 2 (IQR 1–4).

**Table 1:** Diagnosis count using Charlson and Elixhauser co-morbidity lists

Count	Charlson (%)		Elixhauser (%)	
	Cases	Controls	Cases	Controls
0	27.6	36.6	9.2	15.3
1	23.8	23.8	16.9	21.2
2	18.3	16.9	19.1	20.8
3	12.4	10.7	18.4	16.5
4	8.0	5.9	14.1	11.5
5	4.3	3.1	9.7	7.2
> 6	5.6	3.0	12.5	7.5

30-day mortality The EM improved the ability of a logistic regression model (using age and sex as covariables) to predict 30-day mortality (AUROC 0.744 [95% CI 0.727 to 0.760] versus 0.686 [0.668 to 0.705]) among cases. The EM alone performed better than CCI in predicting 30-day mortality (AUROC 0.696 [95% CI 0.677 to 0.714] versus 0.601 [0.582 to 0.619]). Similar findings were observed within the control population: EM + base model AUROC 0.771 (95% CI 0.743 to 0.800) versus EM alone 0.709 (0.678 to 0.740) and EM 0.726 (0.692 to 0.760) versus CCI 0.649 (0.614 to 0.683).

**Fig. 5:**
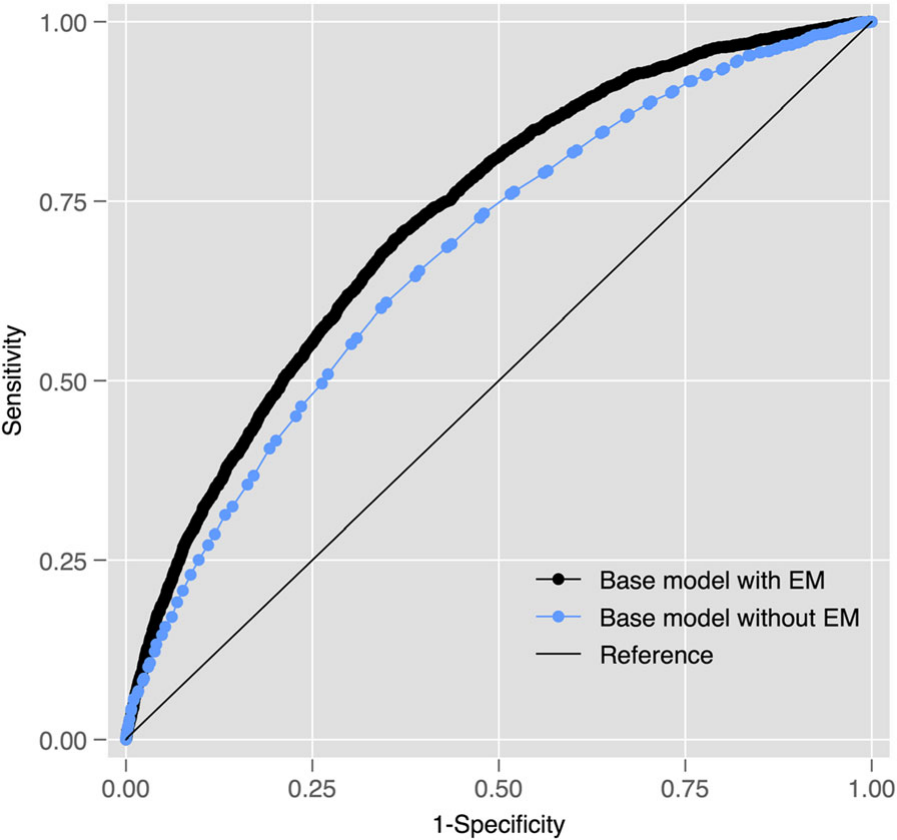
Receiver-Operating Characteristic (ROC) curves for a regression model (co-variables: age and sex) predicting 365-day mortality amongst cases with and without the EM

**Fig. 6:**
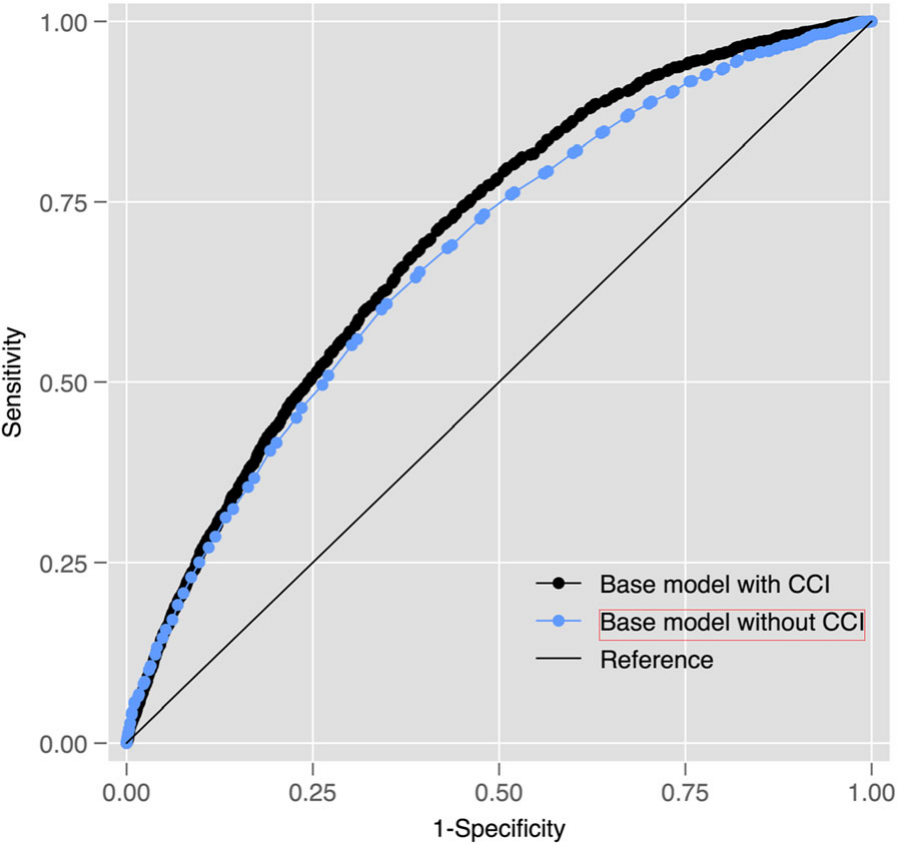
Receiver-Operating Characteristic (ROC) curves for a regression model (co-variables: age and sex) predicting 365-day mortality amongst cases with and without the CCI

365-day mortality Fig. 5 shows that the EM modestly improved the ability of the base regression model to predict 365-day mortality (AUROC 0.726 [95% CI 0.716 to 0.735] versus 0.676 [0.665 to 0.687]) amongst cases. Fig. 6 shows that the CCI only performed marginally better than the base model: AUROC 0.676 [95% CI 0.665 to 0.687]. The EM alone performed better than the CCI (0.672 [95% CI 0.661 to 0.683]) versus 0.611 [95% CI 0.600 to 0.622]). Similar findings were observed within the control population: EM + base model AUROC 0.750 (95% CI 0.740 to 0.759) versus EM alone 0.700 (0.690 to 0.710) and EM 0.696 (0.685 to 0.707) versus CCI 0.635 (0.622 to 0.645).

### Use of diagnoses recorded over different durations

The proportion of patients with a CCI of zero decreased, consistent with expectations, as the length of time over which diagnostic codes were assessed increased, e.g. 64.1% cases had CCI = 0 at 1-year versus 42.9% at 5-year and 27.6% using the complete record. A similar trend was observed for the number of EM co-morbidities, although the difference between 1 and 5 years was less marked (cases 1-year 48.4% versus 5-year 48.3% and complete record 9.2%). The increased capture of diagnostic codes by using the complete primary care record only modestly improved the predictive value of either the CCI (1-year AUROC 0.574 [95% CI 0.555 to 0.572] versus 5-year 0.591 [0.572 to 0.610] versus complete record 0.605 [0.586 to 0.623]) or the EM (1-year 0.645 [0.625 to 0.664] versus 5-year 0.676 [0.657 to 0.696] versus complete record 0.695 [0.677 to 0.714) for 30-day mortality amongst cases. Similar results were observed amongst controls (Additional file 5).

### Use of updated Charlson comorbidity index

The updated CCI described by Quan *et al* [6] performed similarly in this population to the original index, both for 30- (AUROC 0.716 [95% CI 0.699 to 0.733] versus 0.704 [0.686 to 0.721]), and 365-day mortality (AUROC 0.713 [95% CI 0.703 to 7.23] versus 0.700 [0.690 to 0.710]). The modified weights proposed by Quan *et al* [6] have been included as an additional column in Additional file 1.

### Use of Charlson co-morbidities as individual co-variables

The Charlson co-morbidities performed marginally better for predicting 30-day mortality when included as independent co-variables than when used as a single index, both amongst cases (individual co-variables 0.632 [0.612–0.652] versus CCI 0.612 [0.594–0.631]) and controls (0.679 [0.642–0.715] versus 0.660 [0.625–0.693]). This also held for predicting 365-day mortality amongst cases (CM 0.638 [0.627–0.649] versus CCI 0.616 [0.605–0.628]) and controls (individual co-variables 0.662 [0.650–0.674] versus 0.640 [0.628–0.651]).

However, the EM continued to outperform the Charlson co-morbidities in predicting 30-day mortality, even when diagnostic categories were used as individual co-variables (cases: EM 0.695 [0.677–0.714] versus Charlson co-morbidities 0.632 [0.613–0.652] and controls: 0.695 [0.677–0.714) versus 0.632 [0.613–0.652)). The EM also outperformed individual Charlson co-morbidities in predicting 365-day mortality (cases: EM 0.672 [0.661–0.683] versus CM 0.638 [0.627–0.649] and controls: 0.696 [0.685–0.707] versus 0.662 [0.650–0.674]).

## Discussion

The principal aim of this study was to transparently and reproducibly create comorbidity lists for future researchers working with Read-coded databases. The final lists are available as Additional files 1 and 2 that can be readily imported into commonly used statistical software packages (Additional file 1 and Additional file 2). These lists are particularly important for researchers analyzing UK primary care datasets such as Clinical Practice Research Datalink (CPRD) GOLD [15] and The Health Improvement Network (THIN) [16]. Although such researchers may need to include a composite comorbidity score for the purposes of risk adjusting outcomes, it is onerous and time-consuming to create comprehensive code lists for indices such as the EM, which encompasses 31 individual disease categories. This is particularly difficult for Read-coded databases as the Read syntax includes codes along multiple axes, e.g. diseases, procedures, examination findings, and administrative events such as clinic referrals. Read terms also include spelling errors (e.g. “[V]Folow-up exam aft other treatment for malignant neoplasm”, inconsistent abbreviations (“[X]Vit B12/folic/oth ant-megalobl-anaem caus adv ef ther use”, obscure diseases (e.g. “Sequoiosis (red-cedar asthma)”), and synonyms (“Plummer - Vinson syndrome” versus “Plummer-Vinson syndrome”) that can lead to codes being missed. Publicly-accessible lists of diagnostic codes for both the CCI and EM will save analyst time and improve the reproducibility of primary care research.

We have been unable to identify any previous attempts to translate the EM (initially published using ICD-9-CM codes) for use in Read-coded databases. Although the CCI is the most commonly used comorbidity index in studies with administrative data [4], it has less predictive value than the EM in many populations. An earlier study [13] reported such a list for CCI but identified 3156 codes, which is only 54% of those identified by our study. Our study should not be interpreted as criticism of these authors but as an extension of their work as we used their findings – together with those published by other single disease studies – to help create our own CCI list. However, the differences between the two studies highlights the difficulties that research groups face when trying to create comprehensive lists of Read codes and employ them in adequately risk-adjusted research.

This study also showed that the EM performed better than the CCI at predicting hip fracture mortality. However, even the EM only added a modest degree of additional predictive value over and above a simple regression model with age and sex covariables. Although diagnostic codes from the entire lifetime record of patients added predictive value beyond those recorded within the preceding 1- and 5-years, this increase was modest. It is likely that comorbidities recorded within the previous 12-months will be sufficient for risk adjustment in most studies. These findings should reassure researchers that are necessarily working with limited extracts of primary care data.

### Limitations

The main limitation of our study is that it is difficult to be certain that all diagnoses were included within each category. However, we did use a number of strategies to maximize our capture of relevant codes, including screening by independent clinicians and checks using lists created by other researchers for specific disease populations. It is also possible that discrepancies could arise in terms of disease classification. For example, lymphoepithelial carcinoma was categorized as “solid tumour” for the purposes of the EM rather than “lymphoma”. These decisions were aided by recourse to the ICD-9-CM codes used in previous publications as well as textbooks and subject experts. It is, however, possible that some classifications will be contentious or change over time. Although this resource is likely to be sufficient for the purposes of co-morbidity risk adjustment, researchers working on specific disease processes should satisfy themselves that these lists are sufficient for their purposes.

## Conclusion

We have adopted a robust and transparent approach to identifying Read codes that can be used by future researchers to calculate CCI and EM. This study also showed that, although the EM outperforms the CCI and models are improved by using comorbidity codes captured over a long period of time, the differences are modest. Researchers with access to limited datasets concerning comorbidities may create logistic regression models with similar discrimination to those with access to complete healthcare records.

## Additional files

Additional file 1: Read codes for Charlson co-morbidities. (CSV 417 kb)

Additional file 2: Read codes for Elixhauser co-morbidities. (CSV 513 kb)

Additional file 3: Screening Principles. (DOCX 120 kb)

Additional file 4: Read codes used to identify the hip fracture cohort. (DOCX 126 kb)

Additional file 5: Effect of using diagnostic records over different durations. (DOCX 20 kb)

## Data Availability

The data that support the findings of this study are available from the Clinical Practice Research Datalink (CPRD) but restrictions apply to the availability of these data, which were used under license for the current study, and so are not publicly available. Data may however be available on application directly to the CPRD.

## Abbreviations

AUROC: Area under the receiver operating characteristic
CCI: Charlson Comorbidity Index
CI: Confidence interval
CPRD: Clinical Practice Research Datalink
EM: Elixhauser Method
GafREC: Governance arrangements for Research Ethics Committees
GDPR: General Data Protection Regulation
GP: General practice
ICD-9-CM: International classification of disease, 9th edition, clinical modification
IQR: Interquartile range
ISAC: Independent Scientific Advisory Committee
MHRA: Medicines and Healthcare products Regulatory Agency
NHS: National Health Service
ROC: Receiver Operating Characteristic
THIN: The Health Improvement Network
UK: United Kingdom
UTS: Up to standard
