# Supplementary material for: Coding algorithms for defining Charlson and Elixhauser co-morbidities in Read-coded databases
Source: BMC Med Res Methodol. 2019 Jun 6;19:115. doi: 10.1186/s12874-019-0753-5 (PMC6554904; doi:10.1186/s12874-019-0753-5)
Supplement: Supplementary file 3 — Screening Principles. (DOCX 120 kb) [file 12874_2019_753_MOESM3_ESM.docx]

**Additional file 3: Screening Principles**

**Initial Screen**

- The initial search aims to be inclusive and so there should be a rebuttable presumption in favour of each code being included for discussion.
- Use the ICD-9 memoire to suggest search terms but also to delineate the limits of each search.
  - E.g. distinguishing between Charlson “mild liver disease” and “moderate to severe liver disease” codes.
- Include codes that presuppose a diagnosis, even if not truly a diagnostic code.
  - E.g. code “dementia annual review” (6AB..00) as “dementia”.
- Assume that all tests and examination findings are correct.
  - E.g. code “O/E – aortic systolic murmur” (24D5.00) as “valvular heart disease” even if there is considerable scope for misdiagnosis or miscoding.
- Assume that patients being “monitored” for a disease have that disease but do not include codes for “screening”.
  - “Hypertension monitored” (9OIA.11) should be coded as “hypertension” whereas “hypertension screen” (68B1.00) should not.
- Do not include codes that are speculative about potential diagnoses.
  - E.g. do not code “peptic ulcer symptoms” (1956) or “suspected breast cancer” (1J01.00).

**Consensus Meeting**

- Apply the inclusion principles described above in “Initial Screen”.
- Refer to the original Elixhauser and Deyo papers whenever doubt arises about whether or not a code should be included.
  - E.g. "juvenile chronic polyarthritis" (ICD-9 714.3x) is included within "rheumatoid arthritis / collagen vascular diseases" (Elixhauser) but not "rheumatic disease" (Deyo/Charlson).
  - Do not seek to “improve” on the existing Elixhauser and Charlson co-morbidity lists, e.g. by including diseases that are not part of the original scoring systems.
- Use external sources where appropriate to appropriately categorise diseases.
  - E.g. code “reticulosarcoma” (B600.00) as “lymphoma” rather than “solid tumour” after consulting an appropriate pathology resource.
- Apply clinical knowledge to determine whether or not indirect evidence should be treated as diagnostic.
  - E.g. code “seen in hypertension clinic” (9N1y200) as “hypertension” as a diagnosis of hypertension will almost always have been made before a patient is seen in this setting.
  - E.g. do not code “seen in stroke clinic” (9N0p.00) as “cerebrovascular disease” as many patients seen in this setting will end up with an alternative diagnosis, e.g. arteritic anterior ischemic optic neuropathy or Bell’s palsy. “Delivery of rehabilitation for stroke” (7P24200) should however be coded as it is more convincing for a true stroke diagnosis.
- Treat “peripheral vascular disease” as a systemic disease rather than only including codes that strictly relate to peripheral vessels.
  - E.g. Code “aortic aneurysm NOS” (G71z.00) as “peripheral vascular disease”. This is consistent with the approach adopted by Elixhauser (ICD-9 441.4) and Deyo (ICD-9 441.x).
- Do not modify spelling errors or the case of characters in each code.
  - E.g. “[V]Folow-up exam aft other treatment for malignant neoplasm”
- Consensus requires agreement from both screeners. Where agreement cannot be reached, decisions should be escalated to a third clinician.

**Comparison With Existing Code Lists**

- Extract all codes from the Clinical Codes Repository (CCR) that overlap with Charlson and/or Elixhauser categories.
  - E.g. use a list of “moderate or severe liver disease” codes as these are clearly intended to mirror the scoring system proposed by Charlson.
  - E.g. use a list of asthma codes as all should be included within “chronic pulmonary diseases” (Elixhauser).
- Where the same group of authors have uploaded multiple codes to the CCR, only use the most recent available for each co-morbidity.

**Identifying Read Codes That Appear in Multiple Lists**

- Diagnostic code describing multiple discreet pathologies, even if conceptually linked.
  - E.g: “cystic fibrosis related cirrhosis” (C370800).
  - Include in multiple lists (e.g. “respiratory disease” and “mild liver disease”).
- One disease that presupposes another category.
  - “Wernicke's encephalopathy” (C251.11)
  - Include in multiple lists (e.g. “other neurological disorders” and “alcohol abuse”).
- One diagnosis could legitimately be coded in two separate categories.
  - Renal malignant neoplasm (B4A..11)
  - Code in one category – the most clinically appropriate if this can be established (e.g. “solid tumour” rather than “renal disease”).
- Diagnostic code indicating two discreet diseases that are already incorporated within a single Elixhauser category.
  - “Type 1 diabetes mellitus with renal complications” (C108012).
  - Only include within the higher-level diagnostic category (e.g. “diabetes with complications”).
- Miscategorised codes.
  - “Malignant essential hypertension” (G200.00).
  - Categorise appropriately (e.g. hypertension).
- Duplicate codes based on differences in letter capitalization.
  - 9Oj1.00 (“Hypothyroidism monitoring second letter”) and 9OJ1.00 (“Attends asthma monitoring”).
  - Permit both codes.

**Final Check**

- Remove duplicates from each list.
- Check all individual co-morbidities for obvious errors.
