# Supplementary material for: Coding algorithms for defining Charlson and Elixhauser co-morbidities in Read-coded databases
Source: BMC Med Res Methodol. 2019 Jun 6;19:115. doi: 10.1186/s12874-019-0753-5 (PMC6554904; doi:10.1186/s12874-019-0753-5)
Supplement: Supplementary file 4 — Read codes used to identify the hip fracture cohort. (DOCX 126 kb) [file 12874_2019_753_MOESM4_ESM.docx]

**Additional file 4: Read codes used to identify the hip fracture cohort**

| **medcode** | **readcode** | **readterm** |
| --- | --- | --- |
| 5742 | 7K1D000 | Prmy open red+int fxn prox femoral #+screw/nail+plate device |
| 58817 | 7K1D011 | Prim open reduct # neck femur & op fix - Blount nail plate |
| 52395 | 7K1D012 | Prim op red # nck femur & op fix- Charnley compression screw |
| 97337 | 7K1D013 | Prim op red # nck femur & op fix - Deyerle multiple hip pin |
| 94714 | 7K1D014 | Prim open reduct # neck femur & op fix - Holt nail |
| 105352 | 7K1D015 | Prim open reduct # neck femur & op fix - Jewett nail plate |
| 56568 | 7K1D017 | Prim open red # neck femur & op fix - McLaughlin nail plate |
| 46258 | 7K1D018 | Prim open reduct # neck femur & op fix - Neufield nail plate |
| 65536 | 7K1D019 | Prim open reduct # neck femur & op fix - Pugh nail plate |
| 24493 | 7K1D01A | Prim open reduct # neck femur & op fix - Richards screw |
| 57884 | 7K1D01B | Prim open reduct # neck femur & op fix - Ross Brown nail |
| 57889 | 7K1D01D | Prim op red # nck femur & op fix- Zickel intramed nail plate |
| 9792 | 7K1D01E | DHS - Dynamic hip screw primary fixation of neck of femur |
| 12544 | 7K1D01F | Dynamic hip screw primary fixation of neck of femur |
| 33624 | 7K1D600 | Prmy open red+int fxn prox femoral #+screw/nail device alone |
| 34764 | 7K1D700 | Prmy open red+int fxn prox fem #+screw/nail+intramed device |
| 105803 | 7K1DE00 | Prim op red frac neck fem op fix us prox fem nail antirotatn |
| 41888 | 7K1G200 | Primary open reduction+external fixation of femoral fracture |
| 8719 | 7K1J000 | Cls red+int fxn proximal femoral #+screw/nail device alone |
| 53670 | 7K1J011 | Cl red intracaps frac neck femur fix-Garden cannulated screw |
| 40999 | 7K1J012 | Cl red intracaps fract neck femur fix - Smith-Petersen nail |
| 57514 | 7K1J013 | Cls red+int fxn prox femoral #+Richard's cannulat hip screw |
| 35004 | 7K1J500 | Primary int fxn(no red) prox fem #+screw/nail device alone |
| 44594 | 7K1J600 | Primary int fxn(no red) prox fem #+scrw/nail+intramed device |
| 38856 | 7K1J700 | Primary int fxn(no red) prox fem #+screw/nail+plate device |
| 55386 | 7K1JB00 | Primary cls red+int fxn prox fem #+screw/nail device alone |
| 54819 | 7K1JC00 | Prim cls rd+int fxn prox fem #+screw/nail+intramdulry device |
| 46959 | 7K1JD00 | Primary cls red+int fxn prox fem #+screw/nail+plate device |
| 39322 | 7K1Jd00 | Closed reduction of intracapsular # NOF internal fixat DHS |
| 70018 | 7K1K300 | Primary external fixation(without reduction) prox femoral # |
| 102313 | 7K1K500 | Primary cls reduction+external fixation proximal femoral # |
| 6660 | 7K1L400 | Closed reduction of fracture of hip |
| 2225 | S30..00 | Fracture of neck of femur |
| 1994 | S30..11 | Hip fracture |
| 38489 | S300.00 | Closed fracture proximal femur, transcervical |
| 39984 | S300000 | Cls # prox femur, intracapsular section, unspecified |
| 69919 | S300100 | Closed fracture proximal femur, transepiphyseal |
| 65690 | S300200 | Closed fracture proximal femur, midcervical section |
| 52194 | S300300 | Closed fracture proximal femur, basicervical |
| 51861 | S300311 | Closed fracture, base of neck of femur |
| 17019 | S300500 | Cls # prox femur, subcapital, Garden grade unspec. |
| 34351 | S300600 | Closed fracture proximal femur, subcapital, Garden grade I |
| 33957 | S300700 | Closed fracture proximal femur, subcapital, Garden grade II |
| 36599 | S300800 | Closed fracture proximal femur, subcapital, Garden grade III |
| 34078 | S300900 | Closed fracture proximal femur, subcapital, Garden grade IV |
| 45779 | S300A00 | Closed fracture of femur, upper epiphysis |
| 49209 | S300y00 | Closed fracture proximal femur, other transcervical |
| 68229 | S300y11 | Closed fracture of femur, subcapital |
| 62966 | S300z00 | Closed fracture proximal femur, transcervical, NOS |
| 73981 | S301.00 | Open fracture proximal femur, transcervical |
| 50727 | S301000 | Opn # proximal femur, intracapsular section, unspecified |
| 72138 | S301100 | Open fracture proximal femur, transepiphyseal |
| 100771 | S301311 | Open fracture base of neck of femur |
| 38878 | S301500 | Open fracture proximal femur,subcapital, Garden grade unspec |
| 60885 | S301600 | Open fracture proximal femur,subcapital, Garden grade I |
| 67394 | S301700 | Open fracture proximal femur,subcapital, Garden grade II |
| 23803 | S301800 | Open fracture proximal femur,subcapital, Garden grade III |
| 51999 | S301900 | Open fracture proximal femur,subcapital, Garden grade IV |
| 96518 | S301A00 | Open fracture of femur, upper epiphysis |
| 68668 | S301y00 | Open fracture proximal femur, other transcervical |
| 73234 | S301y11 | Open fracture of femur, subcapital |
| 5301 | S302.00 | Closed fracture of proximal femur, pertrochanteric |
| 19117 | S302000 | Cls # proximal femur, trochanteric section, unspecified |
| 19387 | S302011 | Closed fracture of femur, greater trochanter |
| 48337 | S302012 | Closed fracture of femur, lesser trochanter |
| 45141 | S302100 | Closed fracture proximal femur, intertrochanteric, two part |
| 51216 | S302300 | Cls # proximal femur, intertrochanteric, comminuted |
| 8648 | S302400 | Closed fracture of femur, intertrochanteric |
| 44735 | S302z00 | Cls # of proximal femur, pertrochanteric section, NOS |
| 61733 | S303.00 | Open fracture of proximal femur, pertrochanteric |
| 67633 | S303000 | Open # of proximal femur, trochanteric section, unspecified |
| 101567 | S303100 | Open fracture proximal femur, intertrochanteric, two part |
| 97971 | S303300 | Open fracture proximal femur, intertrochanteric, comminuted |
| 39396 | S303400 | Open fracture of femur, intertrochanteric |
| 70479 | S303z00 | Open fracture of proximal femur, pertrochanteric, NOS |
| 28965 | S304.00 | Pertrochanteric fracture |
| 24276 | S30w.00 | Closed fracture of unspecified proximal femur |
| 58642 | S30x.00 | Open fracture of unspecified proximal femur |
| 18273 | S30y.00 | Closed fracture of neck of femur NOS |
| 10570 | S30y.11 | Hip fracture NOS |
| 38054 | S30z.00 | Open fracture of neck of femur NOS |
