# Supplementary material for: Coding algorithms for defining Charlson and Elixhauser co-morbidities in Read-coded databases
Source: BMC Med Res Methodol. 2019 Jun 6;19:115. doi: 10.1186/s12874-019-0753-5 (PMC6554904; doi:10.1186/s12874-019-0753-5)
Supplement: Supplementary file 5 — Effect of using diagnostic records over different durations. (DOCX 20 kb) [file 12874_2019_753_MOESM5_ESM.docx]

**Additional file 5: Effect of using diagnostic records over different durations**

| **Table 1: Charlson Co-morbidity Index (CCI) and 30-day mortality using diagnostic records over different periods (cases)** | | | | | | |
| --- | --- | --- | --- | --- | --- | --- |
| **CCI** | **Diagnoses (1yr look-back)*** | | **Diagnoses (5yr look-back)**** | | **Complete lifetime record***** | |
|  | **Individuals (%)** | **Odds ratio (95% CI)** | **Individuals (%)** | **Odds ratio (95% CI)** | **Individuals (%)** | **Odds ratio (95% CI)** |
| 0 | 8,959 (64.1) | - | 5,994 (42.9) | - | 3,852 (27.6) | - |
| 1 | 3,070 (22.0) | 1.71 (1.45-2.03) | 3,437 (24.6) | 1.60 (1.31-1.94) | 3,320 (23.8) | 1.97 (1.57-2.46) |
| 2 | 1,158 (8.3) | 2.08 (1.67-2.59) | 2,153 (15.4) | 1.96 (1.59-2.40) | 2,558 (18.3) | 1.86 (1.47-2.36) |
| 3 | 449 (3.2) | 1.70 (1.20-2.40) | 1,201 (8.6) | 1.65 (1.28-2.13) | 1,736 (12.4) | 2.37 (1.83-3.06) |
| 4 | 160 (1.1) | 3.06 (1.91-4.90) | 574 (4.1) | 2.36 (1.74-3.19) | 1,117 (8.0) | 2.55 (1.85-3.52) |
| 5 | 40 (0.3) | 3.04 (1.32-7.02) | 277 (2.0) | 1.80 (1.15-2.81) | 602 (4.3) | 3.49 (2.29 -5.31) |
| >6 | 138 (1.0) | 6.25 (3.91-9.98) | 338 (2.4) | 4.46 (3.16-6.29) | 789 (5.7) | 4.31 (2.34-7.95) |

*AUROC 0.574; **0.591; ***0.600

| **Table 2: Charlson Co-morbidity Index (CCI) and 30-day mortality using diagnostic records over different periods (controls)** | | | | | | |
| --- | --- | --- | --- | --- | --- | --- |
| **CCI** | **Diagnoses (1yr look-back)*** | | **Diagnoses (5yr look-back)**** | | **Complete lifetime record***** | |
|  | **Individuals (%)** | **Odds ratio (95% CI)** | **Individuals (%)** | **Odds ratio (95% CI)** | **Individuals (%)** | **Odds ratio (95% CI)** |
| 0 | 19,310 (71.9) | - | 14,192 (52.8) | - | 9,840 (36.6) | - |
| 1 | 4,763 (17.7) | 2.07 (1.51-2.85) | 5,750 (21.4) | 1.93 (1.33-2.81) | 6,404 (23.8) | 2.33 (1.54-3.52) |
| 2 | 1,836 (6.8) | 2.54 (1.71-3.79) | 3,644 (13.6) | 2.35 (1.59-3.48) | 4,527 (16.9) | 2.48 (1.59-3.87) |
| 3 | 682 (2.5) | 2.49 (1.33-4.66) | 1,887 (7.0) | 2.72 (1.73-4.29) | 2,871 (10.7) | 2.65 (1.58-4.44) |
| 4 | 171 (0.6) | 2.95 (1.07-8.12) | 799 (3.0) | 3.28 (1.85-5.82) | 1,588 (5.9) | 4.73 (2.64-8.51) |
| 5 | 56 (0.2) | ¥ | 336 (1.3) | 3.26 (1.39-7.62) | 827 (3.1) | 6.55 (2.85-15.06) |
| >6 | 42 (0.2) | 14.24 (4.27-47.48) | 252 (0.9) | 8.00 (4.02-15.91) | 803 (3.0) | 5.52 (1.29-23.55) |

*AUROC 0.686; **0.637; ***0.646; ^¥^Insufficient deaths in this category.

| **Table 3: Elixhauser co-morbidities and 30-day mortality using diagnostic records over different periods (cases)** | | | | | | |
| --- | --- | --- | --- | --- | --- | --- |
| **ECI** | **Diagnoses (1yr look-back)*** | | **Diagnoses (5yr look-back)**** | | **Complete lifetime record***** | |
|  | **Individuals (%)** | **Odds ratio (95% CI)** | **Individuals (%)** | **Odds ratio (95% CI)** | **Individuals (%)** | **Odds ratio (95% CI)** |
| 0 | 6,767 (48.4) | - | 6,751 (48.3) | - | 1,290 (9.2) | - |
| 1 | 4,415 (31.6) | 1.49 (1.26-1.76) | 4,362 (31.2) | 1.45 (1.22-1.72) | 2,364 (16.9) | 1.85 (1.18-2.90) |
| 2 | 1,910 (13.7) | 1.96 (1.60-2.40) | 1,817 (13.0) | 1.87 (1.51-2.30) | 2,672 (19.1) | 2.50 (1.62-3.85) |
| 3 | 649 (4.6) | 2.70 (2.04-3.57) | 552 (4.0) | 2.58 (1.91-3.49) | 2,567 (18.4) | 2.98 (1.94-4.57) |
| 4 | 176 (1.3) | 3.16 (1.97-5.07) | 121 (0.9) | 2.54 (1.40-4.63) | 1,976 (14.1) | 2.71 (1.75-4.21) |
| 5 | 43 (0.3) | 0.99 (0.23-4.20) | 12 (0.1) | ¥ | 1,355 (9.7) | 3.17 (2.02-4.96) |
| >6 | 14 (0.1) | 3.18 (0.69-14.73) | 359 (2.6) | 3.61 (2.61-5.01) | 1,750 (12.5) | 4.37 (2.85-6.72) |

*AUROC 0.585; **0.595; ***0.604; ^¥^Insufficient deaths in this category.

| **Table 4: Elixhauser co-morbidities and 30-day mortality using diagnostic records over different periods (controls)** | | | | | | |
| --- | --- | --- | --- | --- | --- | --- |
| **ECI** | **Diagnoses (1yr look-back)*** | | **Diagnoses (5yr look-back)**** | | **Complete lifetime record***** | |
|  | **Individuals (%)** | **Odds ratio (95% CI)** | **Individuals (%)** | **Odds ratio (95% CI)** | **Individuals (%)** | **Odds ratio (95% CI)** |
| 0 | 15,661 (58.3) | - | 15,644 (58.2) | - | 4,119 (15.3) | - |
| 1 | 7,428 (27.7) | 1.54 (1.11-2.13) | 7,362 (27.4) | 1.51 (1.09-2.09) | 5,697 (21.2) | 0.99 (0.51-1.94) |
| 2 | 2,738 (10.2) | 2.88 (2.01-4.13) | 2,634 (9.8) | 2.93 (2.04-4.21) | 5,584 (20.8) | 1.53 (0.82-2.83) |
| 3 | 767 (2.9) | 3.20 (1.80-5.67) | 691 (2.6) | 3.04 (1.65-5.61) | 4,428 (16.5) | 1.85 (1.00-3.41) |
| 4 | 221 (0.8) | 3.29 (1.19-9.10) | 150 (0.6) | 2.44 (0.59-1.06) | 3,087 (11.5) | 1.74 (0.91-3.33) |
| 5 | 34 (0.1) | 4.40 (0.59-32.85) | 16 (0.1) | 9.51 (1.22-73.92) | 1,935 (7.2) | 2.99 (1.58-5.66) |
| >6 | 11 (0.0) | ¥ | 363 (1.4) | 3.16 (1.45-6.90) | 2,010 (7.5) | 4.31 (2.35-7.88) |

*AUROC 0.617; **0.645; ***0.660; ^¥^Insufficient deaths in this category.
